# Prevalence and Associated Factors of Diabetes Mellitus among Tuberculosis Patients in South-Eastern Amhara Region, Ethiopia: A Cross Sectional Study

**DOI:** 10.1371/journal.pone.0147621

**Published:** 2016-01-25

**Authors:** Mahteme Haile Workneh, Gunnar Aksel Bjune, Solomon Abebe Yimer

**Affiliations:** 1 Institute of Health and Society, Faculty of Medicine, University of Oslo, Oslo, Norway; 2 Amhara Regional State Health Bureau, Bahir-Dar, Ethiopia; 3 Department of Microbiology, Oslo University Hospital, Oslo, Norway; 4 Department of Bacteriology and Immunology, Norwegian Institute of Public Health, Oslo, Norway; McGill University, CANADA

## Abstract

**Background:**

The association between diabetes mellitus (DM) and tuberculosis (TB) is re-emerging worldwide. Recently, the prevalence of DM is increasing in resource poor countries where TB is of high burden. The objective of the current study was to determine the prevalence and analyze associated factors of TB and DM comorbidity in South-Eastern Amhara Region, Ethiopia.

**Methods:**

This was a facility based cross-sectional study. All newly diagnosed TB patients attending selected health facilities in the study area were consecutively screened for DM. DM was diagnosed based on the World Health Organization diagnostic criteria. A pre-tested semi-structured questionnaire was used to collect socio-demographic, lifestyles and clinical data. Logistic regression analysis was performed to identify factors associated with TB and DM comorbidity.

**Result:**

Among a total of 1314 patients who participated in the study, the prevalence of DM was estimated at 109 (8.3%). Being female [odds ratio (OR) 1.70; 95% confidence interval (CI) (1.10–2.62)], patients age [41–64 years (OR 3.35; 95% CI (2.01–5.57), 65–89 years (OR 3.18; 95% CI (1.52–6.64)], being a pulmonary TB case [(OR 1.69; 95% CI 1.09–2.63)] and having a family history of DM [(OR 4.54; 95% CI (2.36–8.73)] were associated factors identified with TB and DM comorbidity.

**Conclusion:**

The prevalence of DM among TB patients in South-Eastern Amahra Region is high. Routine screening of TB patients for DM is recommended in the study area.

## Introduction

Historically, the association between tuberculosis (TB) and diabetes mellitus (DM) is well known and was of great concern for clinicians and investigators at the dawn of the 20^th^ century. The influence of DM on TB was, however, neglected after the discovery of potent treatment regimens for both diseases [1–4]. Currently, the association between DM and TB is re-emerging worldwide. Cases of DM are increasing in resource poor countries where TB is of a high burden [1–2]. In 2013, there were an estimated 9.0 million incident cases, and 1.5 million TB deaths globally [5]. In the same year, DM affected 382 million people and killed 5.1 million persons. It is projected that the number of people affected by DM will increase to 592 million by 2035 [6] and about 80% of these people live in low and middle income countries where TB is endemic [5–6].

DM triples the risk of developing active TB among infected individuals [7]. It also increases susceptibility to *Mycobacterium tuberculosis* infection and development of diseases. DM directly impairs innate and adaptive immune responses that are necessary to counter the progression from infection to clinical diseases [8]. The association between DM and TB is supported by the fact that patients with DM have impaired cell-mediated immunity, renal failure, micronutrient deficiency and pulmonary microangiopathy, all of which increase their susceptibility to develop TB disease [4, 8–9]. Studies conducted in different parts of the world have shown that 12%-44% of TB diseases were associated with DM [10–15].

Ethiopia is one of 22 high TB burden countries in the world [16] with an estimated incidence and prevalence rates of 210/100000 and 200 /100000 population, respectively [5]. Currently, the country is also facing an increasing rate of DM among its population. With a 4.36% prevalence among its population, Ethiopia is the 3^rd^ highest country in Africa in terms of DM burden [6].

Few studies conducted in Ethiopia reported the magnitude of TB and DM comorbidity [17–19]. However, as these studies were limited to few government hospitals located in major urban areas of some parts of the country, it did not show the burden of the two comorbid conditions among patients from rural areas. It also did not include patients attending private and peripheral health facilities (HFs) where the directly observed treatment short course (DOTS) and DM service are provided. The objective of the current study was to determine the prevalence and associated factors of DM among TB patients attending selected HFs in South-Eastern Amhara Region, Ethiopia.

## Methods

### Study setting

**Fig 1:**
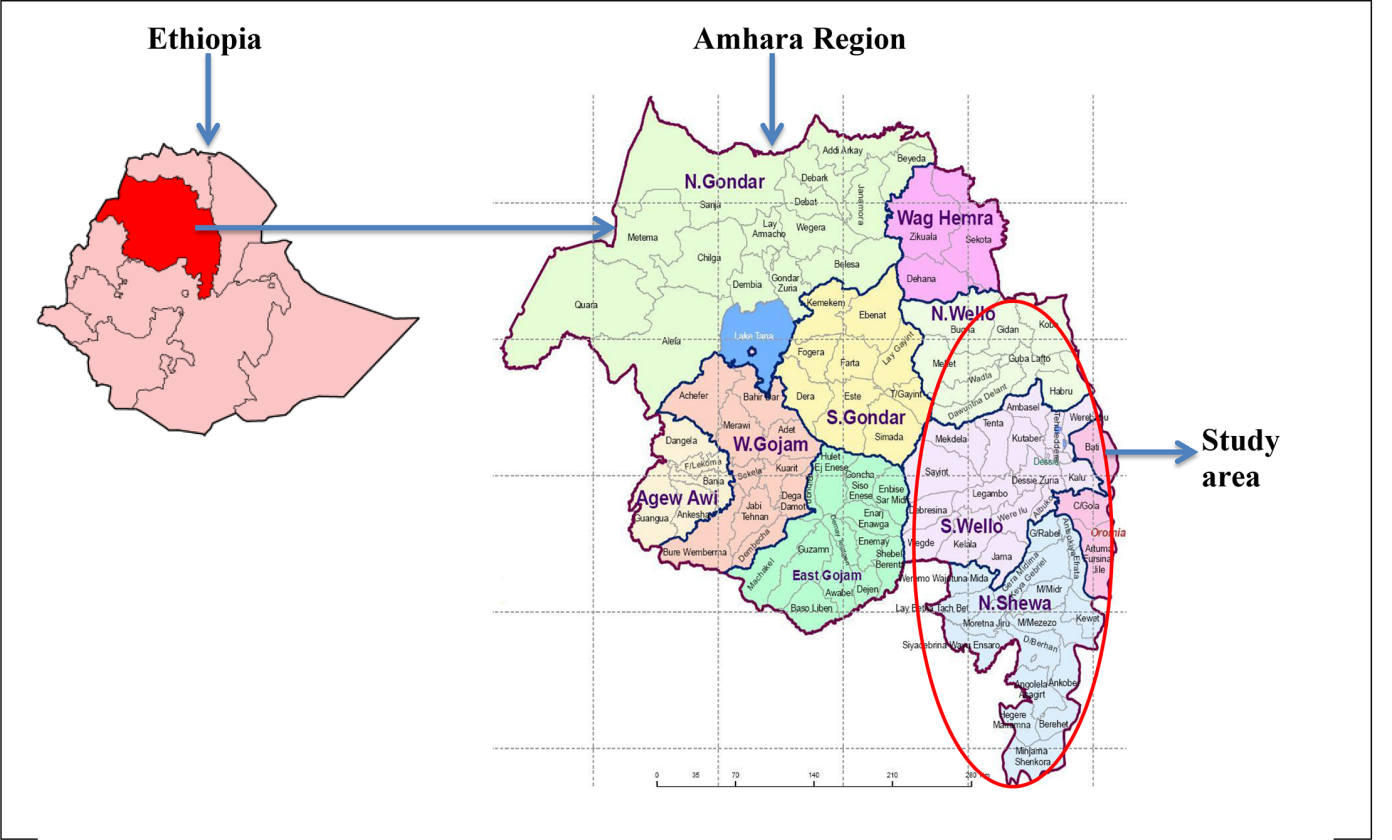
Map of study area (Source: Ethiopia (https://commons.wikimedia.org/wiki/File:Ethiopia-Amhara.png), Amhara Region (https://www.ethiodemographyandhealth.org/Amhara.html)). Accessed on December 29 / 2015.

The study was conducted in South-Eastern part of Amhara Region, Ethiopia. South-Eastern part of Amhara Region consists of four zones and one City Administration namely: North Wollo, South Wollo, North Shewa, Oromia Special Zone and Dessie City Administration (Fig 1).The total population of these zones and City Administration is estimated at 7,358,301. Of these, were 3,684,735 men and 3,673,566 women [20].

### Study design, population and sample size

This was a facility based cross-sectional study which was conducted between September 2013 till September 2014.The study population included all newly diagnosed TB patients aged ≥15 years who were attending DOTS clinics at selected HFs in the study area. Newly diagnosed TB patients aged 15 years and above, transferred in newly diagnosed TB patients who had never started TB treatment in the transferring out HFs and known DM patients who were newly diagnosed for TB were included in the study. Patients less than 15 years of age, "re-treatment" cases, known or suspected multi- drug resistance (MDR) TB cases, patients who were disabled and could not respond to the interview were excluded from the study. The sample size was calculated using the standard formula for estimating a single population proportion, (n) = {[z^2^*p (1-p)]/d^2^}. Therefore, by considering 50% proportion, 95% confidence interval (C.I.) and a margin error of 3%, the sample size was calculated to be 1067. By adding 10% for no response, the total sample size required was 1174 TB patients.

TB diagnosis was made based on guideline developed for the national clinical and programmatic management of TB in Ethiopia. Smear- positive pulmonary TB (PTB) was diagnosed in a patient with at least two sputum smear examinations positive for acid-fast bacilli (AFB) or in a patient with one initial smear examination positive for AFB and radiographic abnormalities consistent with active TB as determined by a clinician. Smear-negative PTB was diagnosed in a patient having symptoms suggestive of TB with at least 3 initial smear examinations negative for AFB by direct microscopy and no response to a course of broad-spectrum antibiotics, and again three negative smear examinations by direct microscopy, and radiological abnormalities consistent with PTB, and decision by a clinician to treat with a full course of anti-TB chemotherapy.The diagnosis of extra PTB (EPTB) was made by identifying AFBs in organs other than lung proven by at least one specimen with confirmed *Mycobacterium tuberculosis* or histological or strong clinical evidence consistent with active EPTB, followed by a decision by a clinician to treat with a full course of anti-TB chemotherapy [16].

All consenting patients were subjected to screening for DM before the start of anti-TB treatment. Screening for DM was done either by random blood sugar (RBS) test right after the arrival of the patient at DOTS unit, and/or fasting blood sugar (FBS) in the morning of the following day after the patient came in fasting state. DM diagnosis was made when RBS test was found to be ≥ 200 mg/dl with the presence of classical sign and symptom of DM and /or FBS test result ≥126 mg/dl at two different times in accordance with the World Health Organization (WHO) DM diagnostic criteria [21]. Self-reporting of having DM was also considered for the diagnosis of DM. Finally, participants were categorized into two groups i.e. TB patient’s comorbid with DM (TBDM) and TB patients not comorbid with DM (TBNDM) based on their DM status. Provider initiated counseling and testing (PICT) service was provided to screen patients for human immuno-deficiency virus (HIV).

### Sampling methods

**Fig 2:**
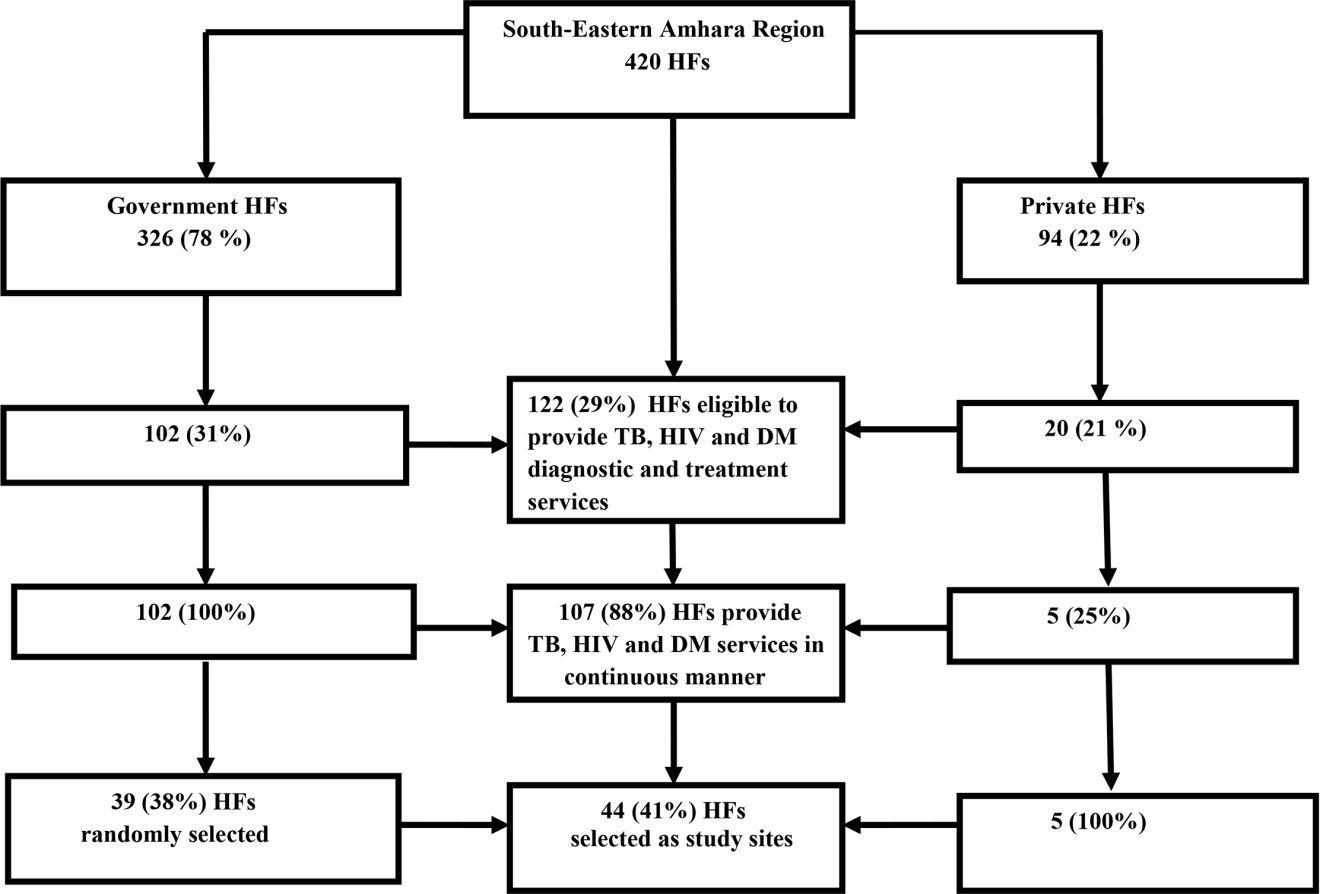
Schematic representation of sampling techniques for selection of study site health facilities (HFs). TB-tuberculosis, HIV-human immuno-deficiency virus, DM-diabetes mellitus.

Random sampling technique was applied to select study sites. There were a total of 420 HFs (326 (78%) government and 94 (22%) private HFs) in the study area. Of these, only 102 (31%) government and 20 (21%) private HFs were eligible to provide TB, HIV and DM diagnostic and treatment services. Among the 20 private HFs, only 5 (25%) provided TB, HIV and DM services in a continuous manner. Fifteen (75%) of the private HFs encountered frequent TB service interruption for various reasons and were excluded. Finally, out of the 102 (31%) government HFs eligible for the study, we randomly selected 39 (38%) HFs. We also added all of the five private HFs that continuously provide TB, HIV and DM services which makes a total of 44 (41%) study sites (Fig 2).

### Data collection and quality assurance

Health workers who provide anti-TB treatment at DOTS unit, laboratory technologist, and a clinician responsible for diagnosis as well as follow up of TB and DM were trained and assigned as data collectors at each study site. Training and pretesting of the questionnaire were conducted at Dessie Health Center (HC). The pre-test result was first discussed and necessary corrections were made on the questionnaire before the actual data collection commenced. The data included soicio-demographic, lifestyle, and clinical parameters including HIV sero-status and body mass index (BMI). To ensure the quality of data, questionnaires were thoroughly checked for completeness and consistency by study supervisors and principal investigator. Manufacturer’s code strips for glucometer and control standard for calibration of auto-analyzer was used to maintain the quality of blood glucose measurement. Internal and external quality control was also ensured for AFB smear microscopy test.

### Ethical approval

The study was initiated after ethical approval was obtained from the Regional Committee for Research Ethics in Norway (REC-Øst, Norway) and Ethiopian Science and Technology Ministry. Permission from Amhara Regional State Health Bureau and from health authorities of the study sites was also received prior to the start of the study. Oral and written information was provided to study participants before informed consent was obtained. Parents/guardians gave consent for study subjects who were between 15 years to 17 years of age. Those TB patients who were found to have DM and HIV were referred to DM and anti-retroviral therapy (ART) clinics for further investigation and appropriate management.

### Statistical analysis

Data entry, cleaning and analysis were performed using Statistical Package for Social Science (SPSS) version 22 Armonk, New York 10504 IBM Corp. Patients with non-conclusive blood glucose test results for DM diagnosis were excluded from the analysis. The main outcome variables were proportions of patients with a diagnosis of TBDM and TBNDM. Categorical variables were expressed as proportions and chi-square analysis was performed to compare proportions. Student’s t test was used to compare means for normally distributed variables. Multivariate logistic regression analysis was performed to analyze the association of predictor variables with the outcome variable. Variables that have a p-value of ≤ 0.2 in the univariate analysis were included in the final model of multivariate analysis. A p-value ≤ 0.05 was considered statistically significant. Acceptance of screening was measured by proportion of participants who consented, and underwent DM screening among those eligible patients invited by the data collectors. The effectiveness of the screening approach was measured using the number of TB patients needed to screen (NNS) to get one additional new DM case and was calculated by the reciprocal proportion of newly detected DM cases.

## Results

### Socio-demographic characteristics of study subjects

Of the total 1353 newly diagnosed TB patients who fulfilled the inclusion criteria, 1335 (98.7%) patients consented and underwent screening. Eighteen (1.3%) patients refused to participate in the screening. Reasons for refusal included lack of money for screening test 15 (1.1%) and fear of losing their blood 3 (0.2%). Among 1335 (98.7%) patients enrolled in the study, 21 (1.6%) did not have confirmatory result for DM and were excluded from the analysis. A total of 1314 participants were included in the final analysis and of these were 692 (52.7%) males and 622 (47.3%) females. The study participant’s age ranged from 15 years to 89 years and the mean age including standard deviation (SD) was 35.74 (±15.26) years. Majority, 928 (70.6%) of the study subjects were in the age range of 15 years to 40 years, were urban residents, had no formal schooling, and had a monthly income of ≤ 18.9 United States Dollar (USD).

### Prevalence of DM among study subjects

The overall prevalence of DM in this study was 109 (8.3%). Of which 45 (3.4%) were known DM patients and 64 (4.9%) were newly diagnosed DM cases. The median duration of DM among known DM patients before TB diagnosis was two years and the highest was four years. The prevalence of DM was higher among patients in the younger age group, urban residents and married individuals (Table 1).

**Table 1:** Socio-demographic back ground of the study participants, South-Eastern Amhara Region, Ethiopia, September 2013—September 2014.

Variables	Type of patients		
	All TB N (%)	TBNDM N (%)	TBDM N (%)
**Total**	**1314**	**1205**	**109**
**Sex**			
Male	692 (52.7)	642 (53.3)	50 (45.9)
Female	622 (47.3)	563 (46.7)	59 (54.1)
**Age in years**			
15–40	928 (70.6)	877 (72.8)	51 (46.8)*
41–64	285 (21.7)	240 (19.9)	45 (41.3)
65–89	101 (7.7)	88 (7.3)	13 (11.9)
Mean (± SD)	35.74 (± 15.26)	35.02 (± 15.06)	43.73 (± 15.33)*
**Residence**			
Urban	826 (62.9)	767 (63.7)	59 (54.1)*
Rural	488 (37.1)	438 (36.3)	50 (45.9)
**Religion**			
Christian	548 (41.7)	508 (42.2)	40 (36.7)
Muslim	766 (58.3)	697 (57.8)	69 (63.3)
**Marital status**			
Single	395 (30.1)	373 (31.0)	22 (20.2)
Married	919 (69.9)	832 (69.0)	87 (79.8)*
**Education**			
No formal schooling	618 (47.0)	558 (46.3)	60 (55.0)
1–6 grade	252 (19.2)	235 (19.5)	17 (15.6)
7–12+1	362 (27.5)	339 (28.1)	23 (21.1)
Diploma and above	82 (6.2)	73 (6.1)	9 (8.3)
**Occupation**			
Unemployed	376 (28.6)	340 (28.2)	36 (33.0)
Student	112 (8.5)	106 (8.8)	6 (5.5)
Self-employed	725 (55.2)	672 (55.8)	53 (48.6)
Government employed	101 (7.7)	87 (7.2)	14 (12.8)
**Monthly income (USD)**^¶^			
No income	167 (12.7)	156 (12.9)	11 (10.1)
≤ 18.9	506 (38.5)	465 (38.9)	41 (37.6)
19–37.9	303 (23.1)	277 (23.0)	26 (23.9)
≥ 38	338 (25.7)	307 (25.5)	31 (28.4)

^¶^ 1USD = 21.1 Ethiopian birr

*P-value ≤0.05

TB- tuberculosis, TBNDM- tuberculosis patient not comorbid with diabetes mellitus, TBDM- tuberculosis patient comorbid with diabetes, SD- standared deviation, USD-United States Dollar

The NNS to find one new cases of DM was 19.8.The NNS was lower among females, patients in the older age group and rural dwellers (Table 2). Of the total 109 (8.3%) DM cases, 93 (85.3%) were identified at HCs, 8 (7.3%) at government hospitals and 8 (7.3%) at private hospitals.

**Table 2:** Number of TB patients needed to screen to find a new case of DM, South- Eastern Amhara Region, Ethiopia, September 2013-September 2014.

Characteristics	Number of TB patient screened for DM	Known DM patients	Patient newly diagnosed for DM	Numbers needed to screen
**Total**	**1314**	**45**	**64**	**19.8**
**Sex**				
Male	692	19	31	21.7
Female	622	26	33	18.1
**Age in years**				
15–40	928	17	34	26.7
41–89	386	28	30	11.9
**Residence**				
Urban	826	25	34	23.5
Rural	488	20	30	15.6
**Marital status**				
Single	395	10	12	32
Married	919	35	52	17
**Education**				
No formal schooling	618	23	37	16.1
1–6 grade	252	9	8	30.3
7–12+1	362	7	16	22.2
Diploma and above	82	6	3	25.3
**Type of TB**				
PTB	770	29	41	18
EPTB	544	16	23	22.9
**Khat chewer**				
Chewer	433	19	24	17
Non-chewer	881	26	40	21.3
**HIV status**				
Positive	261	7	15	16.9
Negative	1045	38	49	20.5
**Family history of DM**				
Yes	68	10	7	8.2
No	1246	35	57	21.2

TB- tuberculosis, DM- diabetes mellitus, PTB- pulmonary tuberculosis, EPTB- extra pulmonary tuberculosis, HIV- human immuno-deficiency virus

### Clinical characteristics

Seven hundred seventy (58.6%) of the study participants were PTB patients, and of these, 420 (54.5%) were smear negative cases. Majority, 70 (64.2%) of TBDM patients were PTB cases. One hundred thirty nine (11.5%) of study participants had impaired fasting glucose (IFG). Mean (±SD) FBS and RBS values were higher among the TBDM group compared to patients in the TBNDM group. Both TBDM and TBNDM groups were equally affected by HIV. Of the 22 (20.2%) HIV infected TBDM comorbid patients, majority, 15 (68.2%) were newly diagnosed DM cases. One hundred forty three (59.8%) TBNDM and 12 (54.5%) TBDM patients were on ART during the study period (Table 3).

**Table 3:** Clinical characteristics of study participants, South-Eastern Amhara Region, Ethiopia, September 2013—September 2014.

Characteristics	Type of patients		
	All TB	TBNDM	TBDM
**Type of TB (n = 1314)**			
PTB	770 (57.6)	700 (58.1)	70 (64.2)
EPTB	544 (41.4)	505 (41.9)	39 (35.8)
**Sputum status (n = 770)**			
Positive	350 (45.5)	322 (46.0)	28 (40.0)
Negative	420 (54.5)	378 (54.0)	42 (60.0)
**FBS (mg/dl) (n = 1207)**			
< 110	972 (80.5)	971 (87.6)	1 (1.0)
110–125	139 (11.5)	137 (12.4)	2 (2.0)
126–650	96 (8.0)	0 (0.0)	96 (97.0)
Mean (± SD)	102.68 (± 37.88)	95.27 (± 12.17)	185.72 (± 91.66)*
**RBS (mg/dl) (n = 107)**			
< 200	99 (92.5)	97 (100.0)	2 (20.0)
≥ 200	8 (7.5)	0 (0.0)	8 (80.0)
Mean (± SD)	125.07 (± 79.98)	104.98 (± 21.38)	320.00 (± 154.58)*
**HIV status (n = 1306)**			
Negative	1045 (80.0)	958 (80.0)	87 (79.8)
Positive	261 (20.0)	239 (20.0)	22 (20.2)
**HIV therapy (n = 261)**			
Pre-ART	106 (40.6)	96 (40.2)	10 (45.5)
ART	155 (59.4)	143 (59.8)	12 (54.5)

*P value ≤0.05

TB- tuberculosis, TBNDM- tuberculosis patient not comorbid with diabetes mellitus, TBDM- tuberculosis patient comorbid with diabetes, PTB-pulmonary tuberculosis, EPTB-extra pulmonary tuberculosis, FBS-fasting blood sugar, RBS-random blood sugar, mg-milligram, dl-decilitre, SD-standared deviation, HIV- human immuno-deficiency virus

### Lifestyle factors

Majority of the participants were found to have never smoked cigarette, were non-alcohol drinkers, did not chew Khat (mild narcotic herb) and had no known contact with a TB patient. Seventeen (15.6%) patients in the TBDM group had a family history of DM compared to 51 (4.2%) of the patients in the TBNDM group. Most of the study participants, 788 (60.0%) had a BMI of <18.5 kg/m^2^. Majority, 6 (5.5%) TBDM patients had a BMI of ≥ 25kg/m^2^ compared to 31 (2.6%) TBNDM patients (Table 4).

**Table 4:** Lifestyle factor among study participants, South-Eastern Amhara Region, September 2013-September 2014.

Characteristics	Type of patients		
	All TB N (%)	TBNDM N (%)	TBDM N (%)
**Total**	**1314**	**1205**	**109**
**Cigarette**			
Never smoke	1195 (90.9)	1093 (90.7)	102 (93.6)
Smoker	119 (9.1)	112 (9.3)	7 (6.4)
**Alcohol**			
Non-drinker	947 (72.1)	867 (72.0)	80 (73.4)
Drunker	367 (27.9)	338 (28.0)	29 (26.6)
**Khat**			
Non-chewer	881 (67.0)	815 (67.6)	66 (60.6)
Chewer	433 (33.0)	390 (32.4)	43 (39.4)
**Contact with TB patients**			
No	1121 (85.3)	1022 (84.8)	99 (90.8)
Yes	193 (14.7)	183 (15.2)	10 (9.2)
**Family history of DM**			
Absent	1246 (94.8)	1154 (95.8)	92 (84.4)
Present	68 (5.2)	51 (4.2)	17 (15.6)*
**BMI(kg/m**^**2**^**)**			
< 18.5	788 (60.0)	729 (60.5)	59 (54.1)
18.5–24.9	489 (37.2)	445 (36.9)	44 (40.4)
≥ 25	37 (2.8)	31 (2.6)	6 (5.5)
Mean (± SD)	18.07 (± 3.01)	18.05 (± 2.94)	18.35 (± 3.72)

* P value ≤ 0.05

TB- tuberculosis, TBNDM- tuberculosis patient not comorbid with diabetes mellitus, TBDM- tuberculosis patient comorbid with diabetes, DM- diabetes mellitus, BMI- body mass index, kg- kilogram, m^2^- meter square, SD- standard deviation

### Factors associated with TBDM comorbidity

Multivariate logistic regression analysis revealed that patients in the TBDM group were more likely to be females [(OR = 1.70; 95% CI 1.10–2.62), 41–64 years of age (OR = 3.35; 95% CI 2.01–5.57), 65–89 years of age (OR = 3.18; 95% CI 1.52–6.64), PTB case (OR = 1.69; 95% CI 1.09–2.63) and having a family history of DM (OR = 4.54; 95% CI 2.36–8.73)] (Table 5).

**Table 5:** Factors associated with TBDM comorbidity, South-Eastern Amahra Region, Ethiopia, September 2013 -September 2014.

Variables	Crude OR (95% CI)	Adjusted OR (95% CI)^¶^
**Sex**		
Male	1	1
Female	1.35 (0.91–1.99)	1.70 (1.10–2.62)*
**Age in years**		
15–40	1	1
41–64	3.22 (2.11–4.93)*	3.35 (2.01–5.57)*
65–89	2.54 (1.33–4.85)*	3.18 (1.52–6.64)*
**Residence**		
Urban	0.67 (0.45–1.00)	0.65 (0.41–1.03)
Rural	1	1
**Education**		
No formal schooling	1	1
1-6grade	0.67 (0.38–1.18)	0.92 (0.49–1.69)
7–12+1	0.63 (0.38–1.04)	1.05 (0.56–1.97)
Diploma and above	1.15 (0.55–2.41)	2.34 (0.96–5.74)
**Marital status**		
Single	1	1
Married	1.77 (1.09–2.86)	0.97 (0.55–1.74)
**Type of TB**		
EPTB	1	1
PTB	**1.29 (0.86–1.95)**	1.69 (1.09–2.63)*
**Khat**		
Non-chewer	1	1
Chewer	1.36 (0.91–2.04)	1.49 (0.97–2.29)
**Family history of DM**		
Yes	4.18 (2.32–7.53)*	4.54 (2.36–8.73)*
No	1	1
**BMI (kg/m**^**2**^**)**		
< 18.5	1	1
18.5–24.9	1.22 (0.81–1.84)	1.47 (0.95–2.27)
≥ 25	2.39 (0.96–5.96)	2.57 (0.91–7.25)

^*^ P value ≤0.05

^¶^—adjusted for sex, age, residence, education, marital status, type of TB, khat chewing, family history of DM and BMI.

TB- tuberculosis, CI-confidence interval, EPTB- extra pulmonary tuberculosis, PTB- pulmonary tuberculosis, DM- diabetes mellitus, BMI- body mass index, kg- kilogram, m^2^-meter square, OR-odds ratio

## Discussion

In this study, we found 8.3% DM prevalence among newly diagnosed TB patients which is higher than the DM prevalence reported in the general population in Ethiopia [6].The finding is in agreement with a previous study reported from the study area [17–18].It is also comparable with the study done in Jammu-India (8.2%) and Uganda (8.5%) [22–23] but lower than that reported from Taiwan (29.5%), Southern-Mexico (29.3%) and Kerela–India (44%) [13–15]. Reasons for the observed variation in prevalence might be related to differences in background between population and screening methods used in DM diagnosis [23–24].

The prevalence of pre-diabetes in our study was 11.5%.This finding is higher than the study done in China (7.8%) [25], Gujarat-India (7%) [26], Saluru–India (8.5%) [27] and Kolar–India (3.1%) [28] but lower than the study finding from Gondar (29.6%), Addis Ababa-Ethiopia (26.7%) and Tamil Nadu–India (24.5%) [18–19, 29].This finding may indicate an increased risk of DM in the future in Ethiopia. Lifestyle change and health promotion activity may delay the onset of DM [25, 27]. The observed DM and prediabetes prevalence in the studied group threatens the gains made in TB control and warrants integrated health services approach to address the burden of the two diseases.

The proportion of new DM cases 64 (4.9%) identified in our study is similar to the study done in India (5%) [30], but higher than from China (3%), Gujarat-India (4%), Saluru–India (3.2%), Kolar–India (2.9%) and Mexico (4.4%) [25–28, 31] and lower than the study finding in Trivandrum-India (7%) [32]. The relatively high proportion of undiagnosed DM in the study may indicate the magnitude of the disease, low awareness, lack of access to DM services, the importance of DM screening among TB patients which is crucial for early diagnosis and treatment of the two comorbid conditions [18–19, 27,31,33].

The number of patients that underwent screening for DM in our study is high and is consistent with the studies done in China [25], Gujarat-India [26] and Saluru–India [27]. HIV counseling and testing service is provided to all TB patients in the study area and this may have helped as an entry point for increased number of DM screening among patients The finding suggests that the screening strategy of TB patients for DM is acceptable and feasible [27].

The NNS to detect a new case of DM among TB patients was 19.8. This number is lower than the finding documented in Gujarat-India, Saluru -India and Kolar–India [26–28] but fivefold higher than the results reported from Kerela-India [15]. The reason for this variation may be due to the high prevalence of DM, the quality of screening test used and the countrywide standard procedure applied for DM screening in India [30]. Given our study findings, it may be more effective to screen specific group of patients including females, patients 41 and above years of age, rural dwellers, married persons, patients who do not have formal education, PTB cases, khat chewers, HIV positive individuals and patients with a family history of DM.

The numbers of DM cases identified among patients attending HCs were higher compared to patients that went to government hospitals and private HFs. This finding is in line with studies done in Saluru–India, Kolar-India, Mexico and Trivandrum-India [27–28, 31–32] but in contrast to the study conducted in India where majority of DM patients were diagnosed at hospitals [30]. The high number of DM patients presenting at periphery level HFs might be related to increased geographical coverage of TB services in the study area. In addition, TB patients who are diagnosis at hospital are usually transferred out to the nearby HFs where they can easily access anti-TB treatments. This finding may indicate the possibility of DM screening at periphery HFs, and the need to access and strengthen DM services where TB services are rendered.

In this study, being female was identified as a risk factor for TBDM comorbidity. This finding is similar with previous studies done in Saskatchewa [34] and Texas-Mexico [35]. But it is in contrast to the study done in Kerela-India [15]. The reasons may be linked to poor health service utilization, caretaking role of women for the sick and influence of estrogen on cytokine production during TB infection. These conditions may increase vulnerability of women to TB and consequently to DM [34].

Study participants in the TBDM group were significantly older than the TBNDM group. This finding is in line with the studies done in Southern-Mexico, Dessie-Ethiopia and Brazil [14, 17, 36]. Increasing age was also identified as one of the risk factors for TBDM comorbidity which is consistent with studies done in Kerela-India [15], Addis Ababa–Ethiopia [19], Tamil Nadu–India [29], Trivandrum–Kerela India [32], China [37] and Puducherry- India [38]. Increasing age is linked to immunosuppression and is one of the risk factor for both TB and DM [2, 6, 17].

Similar to studies done in Malappuram-India [39], Tamil Nadu–India and Trivandrum–Kerela India [29, 32], the prevalence of PTB among patients in the TBDM group was higher than TBNDM group. This might be related to defects in immune functions and decrease capacity of alveolar macrophage to eliminate mycobacterial infection in DM patients [3–4, 9, 40]. The lung is the predominant site of TB diseases among immunocompetent patients [41].

The overall prevalence of HIV (20.0%) is higher than the DM prevalence (8.3%) observed in the study. The finding is in accordance with the study done in Gondar-Ethiopia [18] but in contrast to the study result reported from Malappuram-India [39]. This might be due to the high prevalence of HIV in the study area. Surprisingly, the HIV sero-positivity among both TBDM and TBNDM patient groups was high and is more or less similar with the study done in Addis Ababa-Ethiopia [19].The high number of TB, HIV and DM comorbidity reflects the association of all three diseases and suggests the need for implementing the three-pronged (TB, HIV and DM) collaborative control strategy within the existing health care delivery system. The observed high number of newly detected DM cases among TBHIV comorbid patients may be linked to changes in quality of life and adverse effect of using ART drugs among HIV-positive patients [42]. The high number of DM among HIV positive patients may contribute to increased TB incidence and complication of illness among patients [19]. Routine screening of HIV patients for DM in addition to TB may enhance early detection and better management of DM among TB/HIV coinfected individuals.

The prevalence of TBDM was higher among patients who have a family history of DM. This finding is in line with studies done in Addis Ababa–Ethiopia [19]. Having a family history of DM was among the factors identified that influences the occurrence of TBDM comorbidity. This finding is in line with the study done in Tamilu Nadu-India, China and Puducherry -India [29, 37–38]. Having a family history of DM is known risk factor for DM [6].

The major strength of this study is that it covered urban and rural areas and included government and private HFs at different levels where both DOTS and DM services are simultaneously rendered. Also the enrollment of large number of participants in a consecutive manner reduces selection bias. The following limitations also need worthy of mentioning. We used RBS and FBS tests for making the diagnosis of DM. As these tests have lower sensitivity and specificity compared to oral glucose tolerance test (OGTT) and glycated haemoglobin (HbA1c) [21, 43], we may have underestimated the prevalence of DM among TB patients. In addition, significant number of TB patients whose first DM test result was not conclusive to determine the DM status were excluded from the analysis. This may also contribute to underestimation of the true DM prevalence. TBDM patients who did not visit medical providers for their symptoms may also contribute to underestimation of the magnitude of TBDM comorbid conditions in the study area. We cannot also assess the factors associated for the acceptability of screening because of lack of comparative groups. Social desirability bias due to denial of telling smoking, alcohol drinking and khat chewing habits may affect the study findings related to behavioral risk factors for DM and TB comorbidity.

## Conclusion

The prevalence of DM among TB patients observed in our study is high and may indicate an emerging threat of TBDM comorbidity in Ethiopia in general and South-Eastern Amhara Region in particular. Being female, 41 and above years of age, PTB case and having a family history of DM are important risk factors for TBDM comorbidity. Health education about the importance of getting tested for DM should be given to all TB patients in the study area. Given the rapidly changing economic development in Ethiopia, it may be an appropriate time to think about considering the need for integrated TB and DM service to reduce the burden of the two diseases in the country. In this regard, our study finding may serve as a baseline for larger studies and as a background evidence for policy debate for future possibilities of integrating DM screening into the existing health system in Ethiopia.

## Supporting Information

S1 Dataset Datasets used for the manuscript. (XLS)
